# Race, insurance type, and stage of presentation among lung cancer patients

**DOI:** 10.1186/2193-1801-3-710

**Published:** 2014-12-03

**Authors:** Jimmy T Efird, Hope Landrine, Kristin Y Shiue, Wesley T O’Neal, Tarun Podder, Julian G Rosenman, Tithi Biswas

**Affiliations:** Center for Health Disparities, Brody School of Medicine, East Carolina University, Greenville, NC 27834 USA; Leo Jenkins Cancer Center, Brody School of Medicine, East Carolina University, Greenville, NC USA; Department of Internal Medicine, Wake Forest University School of Medicine, Winston-Salem, NC USA; Department of Radiation Oncology, Seidman Cancer Center, Case Western Reserve University School of Medicine, Cleveland, OH USA; Department of Radiation Oncology, Lineberger Comprehensive Cancer Center, University of North Carolina at Chapel Hill, Chapel Hill, NC USA

**Keywords:** Race, Lung cancer, Disparity, Diagnosis

## Abstract

The purpose of this study was to determine whether African-American lung cancer patients are diagnosed at a later stage than white patients, regardless of insurance type. The relationship between race and stage at diagnosis by insurance type was assessed using a Poisson regression model, with relative risk as the measure of association. The setting of the study was a large tertiary care cancer center located in the southeastern United States. Patients who were diagnosed with lung cancer between 2001 and 2010 were included in the study. A total of 717 (31%) African-American and 1,634 (69%) white lung cancer patients were treated at our facility during the study period. Adjusting for age, sex, and smoking-related histology, African-American patients were diagnosed at a statistically significant later stage (III/IV versus I/II) than whites for all insurance types, with the exception of Medicaid. Our results suggest that equivalent insurance coverage may not ensure equal presentation of stage between African-American and white lung cancer patients. Future research is needed to determine whether other factors such as treatment delays, suboptimal preventive care, inappropriate specialist referral, community segregation, and a lack of patient trust in health care providers may explain the continuing racial disparities observed in the current study.

## Introduction

Lung cancer is the leading cause of cancer deaths in the United States, with mortality rates per 100,000 being higher among African-American (AA) (52.2) than white (49.2) patients (U.S. Cancer Statistics Working Group 2013; Elk and Landrine 2012). While a decline in lung cancer mortality has been observed in the general population, rates remain high in racial and ethnic minorities (Berger et al. 2007). Advanced stage at diagnosis is an important indicator of survival among lung cancer patients. AA lung cancer patients are more likely to be diagnosed at a later stage than whites (Schwartz et al. 2003; Hardy et al. 2009; Halpern et al. 2008). This disparity may be explained by racial differences in health insurance coverage wherein AAs are more likely to be underinsured or have no health insurance (Kirby and Kaneda 2010). Privately insured lung cancer patients have been shown to survive longer than those with Medicaid or no insurance (Biswas et al. 2014). Furthermore, AA patients are less likely to receive preventive cancer services, education, and appropriate specialist referral, and are more likely to experience treatment delays (Felix-Aaron et al. 2005; Esnaola et al. 2008; Bach et al. 1999; Neighbors et al. 2007; Hershman et al. 2009).

In general, cancer patients diagnosed at a later stage have poorer survival than those diagnosed at earlier stages (e.g., 5-year survival rate of 4% among patients diagnosed with late-stage disease compared with 54% among patients diagnosed with early-stage disease (Ward et al. 2010; American Cancer Society 2014). Comparable stage at diagnosis between AA and white lung cancer patients has been observed within the U.S. Military Health System, a single payor system (Zheng et al. 2012; Mulligan et al. 2006). However, to our knowledge, no studies of lung cancer in a civilian population with multiple payors have examined stage of presentation by insurance type. The purpose of this study was to provide further insight into this unanswered question. We hypothesized that AAs, because of multiple historic determinants of poor health, would be diagnosed at a later stage than white lung cancer patients, regardless of insurance type.

## Results

A total of 717 (31%) AA and 1,634 (69%) white lung cancer patients were treated at our center during the study period (Table 1). AA patients were more likely to be younger, male, and receive chemotherapy and radiation therapy. White patients were more likely to receive surgery. AA patients were more likely to have adenocarcinoma and non-small cell lung carcinoma not otherwise specified (NSCLC NOS), and less likely to have squamous cell carcinoma (SCC) and small cell lung carcinoma (SCLC) than whites.

**Table 1:** **Patient characteristics (N = 2,351)**

Patient characteristics	White n (%)	AA n (%)	P-value^†^
Overall	1,634 (70)	717 (31)	-
Age (years)			
Mean ± SD, Median (IQR)	67 ± 10, 68 (14)	64 ± 10, 64 (14)	<0.0001
Sex			
Male	981 (60)	491 (68)	<0.0001
Female	653 (40)	226 (32)	
Histology			
Higher smoking risk	718 (44)	260 (36)	0.0005
SCC	514 (31)	207 (29)	
SCLC	204 (12)	53 (7)	
Lower smoking risk	916 (56)	457 (64)	
Adenocarcinoma	461 (28)	236 (33)	
LCNEC	54 (3)	21 (3)	
Bronchoalveolar	32 (2)	13 (2)	
NSCLC NOS	324 (20)	177 (25)	
Other	45 (3)	10 (1)	
Tobacco use^§‡′^			
Never	139 (9)	58 (8)	0.81
Ever	1,495 (91)	659 (92)	
Surgery			
No	1,066 (65)	549 (77)	<0.0001
Yes	568 (35)	168 (23)	
Chemotherapy			
No	920 (56)	369 (51)	0.031
Yes	714 (44)	348 (49)	
Radiation therapy			
No	984 (60)	391 (55)	0.011
Yes	650 (40)	326 (45)	
Insurance type^‡′^			
Medicare w/o supplement	424 (26)	283 (39)	<0.0001
Medicare with supplement	511 (31)	109 (15)	
Medicaid	92 (6)	105 (15)	
Private insurance	530 (32)	145 (20)	
No insurance/self-pay	77 (5)	75 (10)	

^†^Fisher’s Exact (categorical variables), Exact Deuchler-Wilcoxon (continuous variables). ^§^Cigarette, cigar, pipe, smokeless (snuff, chew).

^‡^Missing values were imputed using the expectation-maximization algorithm (n = 10 simulations).

′Fraction missing information (Tobacco Use = 0.019; Insurance Type = 0.0098).

AA = African-American; LCNEC = large cell neuroendocrine carcinoma; NSCLC NOS = non-small-cell lung carcinoma not otherwise specified; SCC = squamous cell carcinoma; SCLC = small cell lung carcinoma; SD = standard deviation; IQR = Interquartile range.

Adjusting for age, sex, and smoking-related histology, AA patients had a marginally, but statistically significant increased risk (≥14%) of being diagnosed at a later stage (III/IV versus I/II) than whites for all insurance types, with the exception of Medicaid (Table 2). An increasing trend across stage of presentation was observed among AA versus white lung cancer patients, when the data was stratified by age, sex, and smoking-related histology (Table 3). Furthermore, AAs presented with an increasing trend for later stage of lung cancer than whites for Medicare without supplement (adjusted p = 0.015), Medicare with supplement (adjusted p-for-trend = 0.016), and no insurance/self-pay (adjusted p-for-trend = 0.049) (Table 4).

**Table 2:** **Relative risk of an AA patient presenting with Stage III/IV lung cancer by insurance type (N = 2,351)**

Type of health insurance	Stages		Relative risk (95% CI)		
	III/IV	I/II	Unadjusted^†^	Adjusted model 1^†¥^	Adjusted model 2^₰^
	n (%)	n (%)			
All insurance types (n = 2,351)					
White	976 (65)	658 (77)	1.0 Referent	1.0 Referent	1.0 Referent
AA	516 (36)	201 (23)	1.20 (1.13-1.28) p < 0.0001	1.19 (1.12-1.27) p < 0.0001	1.17 (1.10-1.24) p < 0.0001
Medicare w/o supplement (n = 707)					
White	248 (56)	176 (66)	1.0 Referent	1.0 Referent	1.0 Referent
AA	193 (44)	90 (34)	1.17 (1.04-1.31) p = 0.0077	1.16 (1.03-1.29) p = 0.013	1.15 (1.02-1.28) p = 0.018
Medicare with supplement (n = 620)					
White	296 (79)	215 (87)	1.0 Referent	1.0 Referent	1.0 Referent
AA	77 (21)	32 (13)	1.22 (1.06-1.41) p = 0.0061	1.22 (1.05-1.40) p = 0.0073	1.21 (1.05-1.40) p = 0.010
Medicaid (n = 197)					
White	59 (44)	33 (53)	1.0 Referent	1.0 Referent	1.0 Referent
AA	76 (56)	29 (47)	1.13 (0.93-1.37) p = 0.22	1.12 (0.92-1.36) p = 0.24	1.14 (0.94-1.37) p = 0.19
Private insurance (n = 675)					
White	313 (76)	217 (83)	1.0 Referent	1.0 Referent	1.0 Referent
AA	101 (24)	44 (17)	1.18 (1.04-1.34) p = 0.012	1.16 (1.02-1.32) p = 0.024	1.14 (1.005-1.30) p = 0.042
No insurance/self-pay (n = 152)					
White	60 (47)	17 (74)	1.0 Referent	1.0 Referent	1.0 Referent
AA	69 (53)	6 (26)	1.18(1.03-1.35) p = 0.017	1.16 (1.0007-1.33) p = 0.040	1.15 (1.006-1.32) p = 0.041

^†^Relative risk and 95% confidence intervals (CI) computed using Poisson regression with robust variance estimation.

^¥^Adjusted for sex and smoking-related histology.

^₰^Adjusted for age (continuous), sex and smoking-related histology.

AA = African-American.

**Table 3:** **Trend test for lung cancer stage of presentation by age, sex, smoking-related histology (N = 2,351)**

Patient characteristics	Stage				P-for-trend		
	I	II	III	IV	Unadjusted^†^	Adjusted model 1^†¥^	Adjusted model 2^§₰^
	n (%)	n (%)	n (%)	n (%)			
Males (n = 1,472)					<0.0001	<0.0001	0.0005
White	268 (72)	119 (78)	251 (64)	343 (61)			
AA	102 (28)	33 (22)	139 (36)	217 (39)			
Females (n = 879)					0.0009	0.0010	0.0030
White	199 (80)	72 (82)	157 (73)	225 (69)			
AA	50 (20)	16 (18)	57 (27)	103 (31)			
Age < 65 (n = 1,008)					0.0040	0.0147	0.0140
White	142 (67)	73 (78)	176 (63)	247 (58)			
AA	69 (33)	21 (22)	104 (37)	176 (42)			
Age ≥ 65 (n = 1,343)					<0.0001	0.0001	0.0001
White	325 (80)	118 (81)	232 (72)	321 (69)			
AA	83 (20)	28 (19)	92 (28)	144 (31)			
Higher smoking risk (n = 978)^˄^					0.0013	0.0011	0.0015
White	217 (79)	98 (83)	181 (68)	222 (70)			
AA	58 (21)	20 (17)	85 (32)	97 (30)			
Lower smoking risk (n = 1,373)^˅^					<0.0001	0.0001	0.0011
White	250 (73)	93 (76)	227 (67)	346 (61)			
AA	94 (27)	29 (24)	111 (33)	223 (39)			

^˄^Squamous cell carcinoma, small cell lung carcinoma (SCLC).

^˅^Adenocarcinoma, large cell neuroendocrine carcinoma (LCNEC), bronchoalveolar, non-small-cell lung carcinoma not otherwise specified (NSCLC NOS), other histology. AA = African-American

^†^Exact Cochran-Armitage trend test.

^§^Likelihood ratio trend test.

^¥^Adjusted for sex and smoking-related histology, unless a stratifying variable.

^₰^Adjusted for age (continuous), sex and smoking-related histology, unless a stratifying variable.

**Table 4:** **Trend analysis for lung cancer stage of presentation by insurance type (N = 2,351)**

Type of health insurance	Stage				P-for-trend		
	I	II	III	IV	Unadjusted^†^	Adjusted model 1^†¥^	Adjusted model 2^₰§^
	n (%)	n (%)	n (%)	n (%)			
All insurance types					<0.0001	<0.0001	<0.0001
White	467 (75)	191 (80)	408 (68)	568 (64)			
AA	152 (25)	49 (20)	196 (32)	320 (36)			
Medicare w/o supplement					0.0079	0.014	0.015
White	129 (67)	47 (64)	107 (58)	141 (55)			
AA	64 (33)	26 (36)	78 (42)	115 (45)			
Medicare with supplement					0.011	0.013	0.016
White	156 (88)	59 (84)	118 (80)	178 (79)			
AA	21 (12)	11 (16)	29 (20)	48 (21)			
Medicaid					0.48	0.55	0.44
White	22 (48)	11 (69)	19 (41)	40 (45)			
AA	24 (52)	5 (31)	27 (59)	49 (55)			
Private insurance					0.049	0.10	0.14
White	149 (80)	68 (92)	139 (78)	174 (74)			
AA	38 (20)	6 (8)	39 (22)	62 (22)			
No insurance/self-pay					0.016	0.047	0.049
White	11 (69)	6 (86)	25 (52)	35 (43)			
AA	5 (31)	1 (14)	23 (48)	46 (57)			

^†^Exact Cochran-Armitage trend test.

^§^Likelihood ratio trend test.

^¥^Adjusted for sex and smoking-related histology.

^₰^Adjusted for age (continuous), sex and smoking-related histology.

AA = African-American.

## Discussion

Our results support previously published findings that AA lung cancer patients are more likely to be diagnosed at a later stage than whites (Yang et al. 2010; Schwartz et al. 2003; Hardy et al. 2009; Halpern et al. 2008). Additionally, it is uniquely shown that AA lung cancer patients with similar insurance coverage were diagnosed with more advanced disease than whites, except for Medicaid patients. This finding suggests that equivalent insurance coverage may not ensure equal presentation of stage between AA and white lung cancer patients.

The impact of race on stage at diagnosis may be related to factors other than insurance type in the current study. For example, social exclusion and provider mistrust may affect health outcomes for AA patients regardless of socioeconomic position (Carpenter et al. 2009; Hausmann et al. 2008; Williams and Jackson 2005). Other factors such as socioeconomic status (SES), residential segregation, and area-level SES also have been shown to contribute to lung cancer prevalence rates, stage at diagnosis, quality of care, and survival rates (Bennett et al. 1998; Hayanga et al. 2013; Landrine et al. 2012; Smith et al. 2012; Hao et al. 2011b, [a]). However, the extent to which racial differences in stage at diagnosis of lung cancer patients is attributable to the above social determinants, as well as health beliefs, knowledge and behavior, remains unclear.

In the current study, stage of presentation did not statistically differ between AA and white lung cancer patients with Medicaid coverage. Possibly, Medicaid represents a population in which outcomes are equalized regardless of race. Medicaid patients presumably have low SES and equally poor access to care due to low provider acceptance of this plan (Forrest et al. 2007). In contrast, patients who did not have health insurance may reflect a marginally higher socioeconomic position (e.g., working poor) closer to non-Medicaid coverage. Alternatively, this finding may represent an unexplained paradox, specific to our patient population, or chance.

Our findings support earlier research demonstrating that AA lung cancer patients are less likely to receive surgery for their disease compared with whites (Bach et al. 1999; Hardy et al. 2009; Yang et al. 2010; Said et al. 2010). A possible explanation for this finding is that AA patients are more likely to present with advanced disease that is not surgically treatable. In general, it is well known that minorities prioritize health to a lesser degree, postpone seeing a doctor, or do not have a regular physician, which may have contributed to the later stage at presentation observed in AA patients.

Lung cancer mortality rates per 100,000 in our region are consistently higher among male (89.6) and female (45.5) AAs than male (82.7) and female (35.7) whites (Rao and Knight 2008). We observed a similar trend for advanced stage malignancy upon presentation for both male and female AA patients. Furthermore, a statistically significant trend for later stage at presentation was observed by age of Medicare eligibility (i.e., <65 vs. ≥65). While smoking causes all types of lung cancer, the percentage of cases attributable to smoking varies by histology, with squamous and small cell carcinomas conveying the greatest risk (Khuder 2001; Barbone et al. 1997; Boffetta et al. 2011). However, AAs in our study presented with more advanced stage lung cancer than whites, regardless of smoking-related histology.

Currently, no recommendations exist to effectively screen for lung cancer by race in the general population (Detterbeck et al. 2013; U.S. Preventive Services Task Force 2014; Aberle et al. 2013). The “fundamental cause of disease approach” argues that when no basic screening tool exists, racial differences in early detection and disease severity at presentation may be less pronounced (Kim et al. 2010). However, we observed that AA patients presented with more advanced stage lung cancer overall and within insurance type compared with whites. An effective screening tool, if it were to exist for AAs, likely would magnify this observed health disparity. Accordingly, AAs constitute a high-risk group who should be appropriately targeted for screening as new and effective technologies for identifying lung cancer are developed and made available (Gareen et al. 2014; Black et al. 2014).

This study has several strengths. A large AA population in eastern North Carolina allowed for us to report on a group that has experienced historic differences in access to care and discrimination. Ninety percent of the counties in this region have a higher percentage of AAs than the national value of 13.1% (United States Census Bureau 2010; Efird et al. 2013). Our rural catchment area represents a unique population regarding health care resources. For example, residents of rural regions have limited access to medical providers compared with urban areas (Shugarman and Farley 2003; Pathman et al. 2006). Data also were collected from a population-based cancer registry with a standardized data entry system and routine quality control.

This study was limited by the incompleteness of some variables (n = 45, tobacco use; n = 21, payor status). However, the relative imputation efficiency for these variables exceeded 99.8% and it is unlikely that this would have biased our results. Important pieces of information that would have been useful in the etiologic and explanatory interpretation of the study findings (e.g., income, education and occupation, individual-level SES, marital status, evidence of doctor’s or emergency room visits within the year prior to cancer diagnosis, percentage of patients with an x-ray obtained within 6 months prior to diagnosis, pack years smoked, and age of first tobacco use) were not available in the current analysis. Ideally, future studies will be designed to collect this information.

Our analysis by insurance type and stage resulted in small sample sizes within groups. Insurance status was recorded at the time of diagnosis and it also is possible that Medicaid was retroactively granted to uninsured or underinsured patients as a result of diagnosis. The latter limitation may have resulted in misclassification and selection bias. Self-reports of tobacco use have been shown to be lower than actual use, especially among AA patients (Gorber et al. 2009; Corral et al. 2013; Fisher et al. 2008), and the tobacco products that AAs are more likely than whites to smoke (i.e., cigarillos) rarely are assessed (Corral et al. 2013). The reporting of tobacco use is a sensitive and potentially stigmatizing topic and some patients may have been reluctant to report usage truthfully. However, any resulting bias likely had a nominal impact on our results since the vast majority of lung cancer patients have a history of tobacco use. The absence of individual and area-level socioeconomic measures was another potential limitation of our analyses.

The results of this study are from a rural region with a unique population and may not generalize to other areas of the country. However, because our data were collected from one health system, this might have partially controlled for other healthcare-related factors (e.g., variation in misclassification of staging system and payor status, as well as geography). Furthermore, we were unable to reliably estimate socioeconomic position using zip codes because a large percentage of patients in our region have postal box addresses. However, eastern North Carolina is relatively homogenous with respect to socioeconomic status and it is unlikely that inclusion of this variable in our models would have substantively changed the results of this study.

## Conclusions

Equality in health insurance (a crude measure of access to care) may not ensure equal presentation of stage between AA and white lung cancer patients. Further research is needed to identify the underlying determinants and appropriate measures for ameliorating this disparity.

## Methods

### Patients and clinical variables

Patients who were diagnosed with lung cancer between 2001 and 2010 at the Leo Jenkins Cancer Center, East Carolina University were included in this study. Approval for the study was obtained from the Institutional Review Board at the Brody School of Medicine.

Methodology for data collection has been previously described (Biswas et al. 2014; Elchoufani et al. 2013). Briefly, data were obtained from the Vidant Medical Center Cancer Registry, which includes patients seen at Vidant Medical Center, Brody School of Medicine, Physicians East, SurgiCenter, and other local medical clinics. The registry follows standard data collection and validation procedures and has received the Commission on Cancer Outstanding Achievement Award from the American College of Surgeons. When necessary, information in our tumor registry was verified by cross-linkage with administrative billing records.

Our analysis data set included information on age, sex, race, stage, histology, tobacco use, and treatment history (surgery, chemotherapy, and radiation therapy according to current standards of practice). Lung cancer was categorized into 7 groups based on pathology reports and included SCC, adenocarcinoma, NSCLC NOS, SCLC, large cell neuroendocrine carcinoma (LCNEC), bronchoalveolar carcinoma, and other histology. Histology was stratified by high (SCC, SCLC) and low (adenocarcinoma, LCNEC, bronchoalveolar, NSCLC NOS, other) smoking risk (Khuder 2001; Barbone et al. 1997; Boffetta et al. 2011). Tumor stage at initial diagnosis/presentation was categorized according to criteria established by the American Joint Committee on Cancer (AJCC). Health insurance coverage was determined at the time of diagnosis and defined as Medicare with and without supplement, Medicaid, private, and no insurance/self-pay. Insurance was coded as a single variable field. Individuals with Medicare may have had supplemental insurance, and this was coded as a separate category from Medicare without supplement. Because of small numbers, patients with TriCare (n = 26) or Veteran’s Affairs insurance (n = 3) were recoded as private insurance and Medicare without supplement, respectively. Tobacco use was self-reported and included cigarette, cigar, pipe, and smokeless tobacco (snuff, chew) use. Similarly, information on age, sex, and race was provided by the patient.

### Statistical analysis

Categorical variables were reported as frequency and percentage while continuous variables were reported as mean, ± standard deviation, median, and interquartile range. Statistical significance for categorical variables was tested using the Fisher’s exact test and the Deuchler-Wilcoxon procedure for continuous variables. Relative risks with 95% confidence intervals (CI) were used as the measure of association between predictor variables and stage at presentation (III/IV versus I/II) and were computed using a robust Poisson regression model. Trend across levels of stage by race was assessed using the exact Cochran-Armitage or likelihood ratio test for trend.

An iterative expectation-maximization (EM) algorithm was used to impute missing values (Dempster et al. 1977; Little et al. 2012; Ware et al. 2012). The relative efficiency for each imputed variable (tobacco use, insurance type) exceeded 99.8%. Model goodness of fit was assessed using the Hosmer-Lemeshow test (Hosmer and Lemeshow 2000). Rounding was performed using the method of Holly and Whittemore (Holly et al. 1989). Statistical significance was defined as p < 0.05. SAS Version 9.4 (Cary, NC) was used for all analyses.

## Abbreviations

AA: African-American
AJCC: American Joint Committee on Cancer
CI: Confidence intervals
EM: Expectation-maximization
IQR: Interquartile range
LCNEC: Large cell neuroendocrine carcinoma
NSCLC NOS: Non-small cell lung carcinoma not otherwise specified
RR: Relative risk
SCLC: Small cell lung carcinoma
SD: Standard deviation
SES: Socioeconomic status
SCC: Squamous cell carcinoma.
